# Anderson–Fabry Disease: Focus on Ophthalmological Implications

**DOI:** 10.3390/life14121531

**Published:** 2024-11-22

**Authors:** Francesca Giovannetti, Mattia D’Andrea, Federico Bracci, Andrea Frustaci, Cristina Chimenti, Pietro Mangiantini, Alessandro Lambiase, Marco Marenco

**Affiliations:** 1 Rare, Degenerative and Inflammatory Ocular Diseases Unit, Department of Sense Organs, La Sapienza University, Viale del Policlinico 155, 00161 Rome, Italy; francesca.giovannetti@uniroma1.it (F.G.); mattia.dandrea@uniroma1.it (M.D.); federico.bracci@uniroma1.it (F.B.); pietro.mangiantini@uniroma1.it (P.M.); marco.marenco@uniroma1.it (M.M.); 2 Cellular and Molecular Cardiology Lab, IRCCS L. Spallanzani, Via Portuense, 292, 00149 Rome, Italy; biocard@inmi.it; 3 Department of Clinical, Internal, Anesthesiologist and Cardiovascular Sciences, La Sapienza University, Viale del Policlinico 155, 00161 Rome, Italy; cristina.chimenti@uniroma1.it

**Keywords:** Fabry disease, lysosomal storage disorder, cornea verticillata, enzyme replacement therapy, vessel tortuosity, retinal vessel density, confocal microscopy, angio-OCT

## Abstract

Fabry disease (FD) is a rare X-linked lysosomal storage disorder with a broad spectrum of clinical manifestations, including severe complications, such as end-stage renal disease, hypertrophic cardiomyopathy, and cerebrovascular disease. Enzyme replacement therapy (ERT), when initiated early, has been shown to reduce the incidence of severe events and slow disease progression. In the classic form, characterized by the absence of α-galactosidase A (α-Gal A) enzyme activity, diagnosis is straightforward. However, when residual activity is present, the delayed and less obvious presentation can make diagnosis more challenging. Ophthalmological alterations, which can be detected through non-invasive examinations may play a crucial role in correctly assessing the patient in terms of diagnosis and prognosis, particularly in these atypical cases. Recognizing these ocular signs allows for timely intervention with ERT, leading to improved patient outcomes. This review highlights the importance of ophthalmological findings in FD, emphasizing their role in diagnosis and treatment planning. By raising awareness among ophthalmologists and healthcare specialists, this review aims to improve disease management, offering tools for early detection and better long-term prognosis in patients with FD.

## 1. Introduction

Fabry disease (FD) is a rare, X-linked, lysosomal storage disorder characterized by an estimated prevalence of the classical form ranging from 1:476,000 to 1:117,000 live male births. The prevalence of the non-classical form is likely much higher, especially in women, due to the broader variability and subtler presentation of clinical symptoms [1]. FD is caused by various mutations in the α-galactosidase A (α-Gal A) gene, which is located on the X chromosome at the q22.1 region [2]. As a result, there is reduced or absent activity of α-galactosidase A enzyme, leading to the slow and progressive accumulation of glycolipids in plasma and various tissues throughout the body, including smooth muscle and the corneal epithelium. The primary substances that accumulate are globotriaosylceramide (GL-3, Gb-3) and globotriaosylsphingosine (lyso-GL-3), in their deacylated forms [3]. The mechanisms driving the disease are intricate and remain incompletely understood, as for many lysosomal storage disorders, but autophagy dysfunction plays the key role in the process [4].

**Figure 1:**
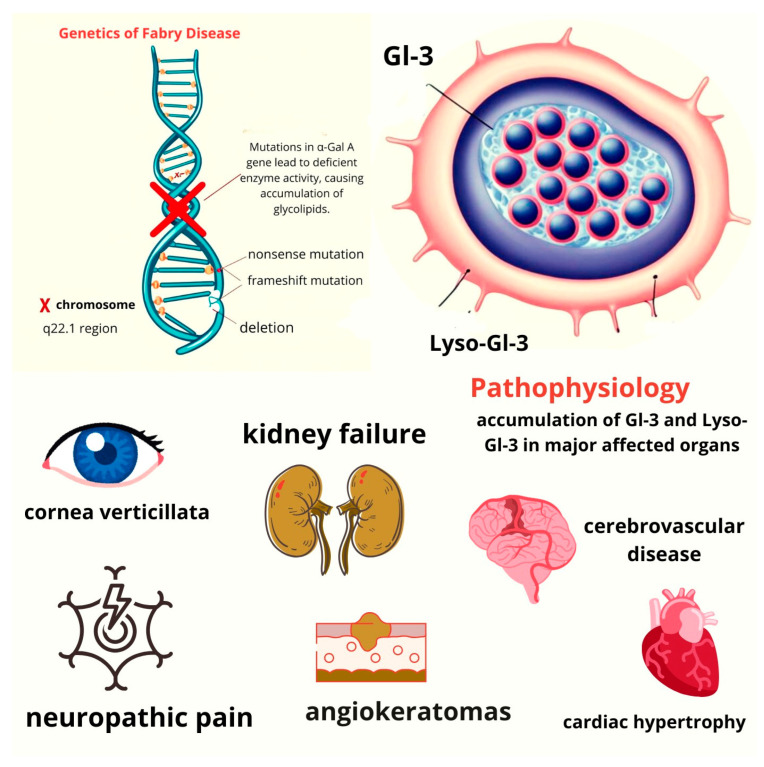
Top left: illustration of X-linked inheritance in Fabry disease, showing various mutations that can affect the q22.1 region of the X chromosome (missense, frameshift, deletion, etc.). **Top right**: cell filled with Gl-3 and lyso-Gl-3 accumulation. **Bottom left and right**: schematic overview of Fabry disease pathophysiology, highlighting how the accumulation of Gl-3 and lyso-Gl3 leads to major organ dysfunction.

This storage disorder presents with a wide range of clinical symptoms, with the most severe being end-stage renal disease, hypertrophic cardiomyopathy, and cerebrovascular disease [5] (Figure 1).

### 1.1. Clinical Phenotypes Related to α-Gal Activity and Genetic Patterns

Symptoms of Fabry disease typically appear during childhood, with patients potentially progressing to organ failure and eventual death [6].

The “classic” phenotype of Fabry disease is observed in males with either absent or severely reduced α-Gal A activity, less than 1% of mean normal activity. This leads to a significant accumulation of GL-3 in endothelial cells, smooth muscle cells, cardiomyocytes, and podocytes [6].

α-Gal A is a homodimeric glycoprotein, and mutations in the gene located on the long arm of the X chromosome can affect its production. Nonsense mutations, consensus splice site mutations, frameshift mutations, and deletions are the most impactful, causing a severe reduction in or absence of enzyme activity and resulting in the classic phenotype. In contrast, missense mutations are associated with a “later-onset” phenotype due to residual enzyme activity. A recent newborn screening study showed that the classic phenotype is less common than the later-onset form, with a prevalence of 1 in 22,570 males affected by the classic form compared to 1 in 1390 for the later-onset form [6].

In heterozygous women, the disease can manifest in various degrees of severity. The variability in signs and symptoms is influenced by both the type of mutation and the level of expression of the muted X chromosome. The process of random inactivation of one X chromosome, known as lyonization, determines the clinical phenotype variability in heterozygous women. When the disease-causing X chromosome is predominantly expressed, signs and symptoms resemble those of males. However, if the disease-causing X chromosome is largely inactivated, the female patient may experience mild symptoms or be asymptomatic. Therefore, assessing the X chromosome inactivation pattern can help predict the future clinical severity of the disease [7,8].

A wide range of GLA mutations have been reported, and ongoing studies are associating these mutations with phenotypes and distribution patterns. Up-to-date databases are available on online platforms for reference [9,10,11,12].

### 1.2. Classic Clinical Pattern

Classic FD develops during childhood, with chronic neuropathic pain and episodic severe pain crises associated with hypohidrosis and heat/cold intolerance, angiokeratomas, gastrointestinal disturbances, and cornea verticillata [13].

Neuropathic pain results from small nerve fiber loss due to GL-3 storage in dorsal root ganglia leading to upregulation of Na+ channels and axon degeneration due to ischemia. Angiokeratoma is caused by weakened capillary walls and ectasia from GL-3 accumulation. This process also leads to retinal and conjunctival vascular tortuosity. Gastrointestinal symptoms, such as nausea, vomiting, abdominal pain, constipation, and diarrhea, are due to the narrowing of mesenteric blood vessels, visceral ischemia, and GL-3 accumulation in autonomic ganglia.

Occult kidney injury may occur at a young age because of podocyte damage, leading to albuminuria and glomerulosclerosis [14,15]. Chronic kidney disease (CKD) can develop early, with late-stage manifestations including left ventricular hypertrophy, arrhythmias, and hearing loss. Cardiovascular disorders result from accumulation of GL-3 in cardiac tissue, which triggers an inflammatory response, finally leading to cellular dysfunction. Arrhythmias, transient ischemic attacks (TIAs), and strokes can lead to premature death, especially when treatment is delayed [16,17,18,19,20,21].

Glycolipids storage in lymphatic vessels causes lymphedema, which may be localized peri-orbitally or present as pitting edema.

### 1.3. Non-Classical Clinical Pattern

Patients with a later-onset pattern exhibit a slower disease progression due to residual GLA function: cardiac symptoms and CKD typically manifest between the fourth and the seventh decades of life. Heterozygous female patients display a wide spectrum of clinical presentations, ranging from asymptomatic to mild or later-onset phenotypes, often affecting one or a few organs. Although rare, the severe phenotype complicated with severe multiorgan involvement can still occur in heterozygous females even at a relatively young age [22].

### 1.4. Diagnosis

To diagnose Fabry disease in men, an α-Gal activity test is required. However, to predict or establish the clinical phenotype, identifying the specific disease-causing mutation is essential. In female patients, residual enzymatic activity that can often be sufficient and demonstrating one α-Gal mutation on X chromosome is necessary. Enzymatic activity can be measured on dried blood spots (DBSs), in plasma or in leukocytes [5].

When a novel mutation (known as “variant of unclear significance”, VUS) occurs in a patient with clinical or histopathological evidence of FD, it is necessary to determine the role of the mutation itself with an in vitro GLA mutation expression assay. While clinical features are valuable for diagnosis, elevated plasma or urinary GL-3 and plasma lyso-GL-3 (globotriaosylsphingosine) are more telling markers. Lyso-GL-3 in plasma is a highly sensitive biomarker, especially for diagnosing the classic form of the disease, whereas normal values often indicate a benign variant. Therefore, this assessment should be considered for prognosis in cases of VUS [23,24].

When typical signs, such as kidney function impairment, neuropathic pain, cerebral manifestations, or cardiac symptoms, are absent, it is useful to verify the presence of cornea verticillata, a history of hand and foot pain that developed during childhood, or angiokeratomas localized in the trunk area, umbilicus, and perioral region, as these signs can support the diagnosis [25].

### 1.5. Management

Managing FD patients effectively requires strict follow-up to prevent clinical progression and irreversible tissue damage. To assess FD, a system of scoring based on signs and symptoms called the Mainz Severity Score Index (MSSI) is applied. It consists of four sections: general, neurological, cardiovascular, and renal. Each section includes signs and symptoms related to FD, weighted according to their contribution to morbidity. The scores of each section are then summed to calculate the total MSSI score: <20 indicates mild disease, between 20 and 40 moderate disease, and >40 indicates severe disease [1].

The main treatment available since 2001 is enzyme replacement therapy (ERT) with agalsidase alpha and beta [26,27,28,29].

It is now well known that early initiation of treatment decreases the incidence of severe events [30]. According to the European Fabry Working Group, ERT should be considered in symptomatic and asymptomatic males with the classic phenotype before 16 years old. Non-classic males and females should start treatment as soon as clinical signs appear, or if asymptomatic, in the presence of biochemical (GFR > G2, persistent albuminuria > 30 mg/g), histological (moderate or severe GL-3 inclusions), or imaging evidence of FD, such as left ventricular hypertrophy (LVH) and myocardial fibrosis on cardiac MRI, or pulmonary infiltrations or fibrosis visible on chest X-ray or CT [31]

Conversely, treatment should not be started in patients with end-stage FD, end-stage renal insufficiency (GFR < 45 mL/min/1.73 m), heart failure (NYHA class IV), or with a life expectancy of less than one year [31].

It is important to note that IgG antibodies against agalsidase alpha and beta are relatively common, especially in patients with a total absence of α-Gal A. For this reason, periodic monitoring of antibody status is recommended, even though the impact of these antibodies on treatment effectiveness remains unclear [32].

Pegunigalsidase alpha and moss-aGal, two plant-derived enzymes, have been recently proposed for ERT with promising results. Pegunigalsidase alpha has been recently approved by regulatory agencies [33].

In 2016, a novel therapeutic option was introduced: migalastat, an orally administered pharmacological chaperon that improves a-Gal activity by binding the enzyme’s active site. Long-term data are still lacking, and this treatment is only suitable for selected patients with “amenable” mutations [34].

A new oral therapy explored for patients with limited enzymatic activity is represented by substrate reduction therapy (SRT). SRT has been proven beneficial for Gaucher disease. The aim is to block glucosylceramide synthase, reducing Gb3 and other glycosphingolipids, offering an alternative treatment for patients regardless of GLA mutation type.

New therapeutic options are underway to treat FD. Early bone marrow transplants showed partial or complete symptom reversal, but carry risks, particularly for slowly progressing diseases like Fabry, where complications (e.g., graft vs. host disease) may outweigh benefits. Recent Fabry gene therapies using lentiviral vectors (AVR-RD-01) or adeno-associated (AAV) viral vectors have shown promise in enzyme overexpression in plasma, though immune responses and efficacy in all affected tissues remain challenges. mRNA therapies offer transient yet potentially safer effects, requiring repeat dosing but enabling enzyme production without insertional mutagenesis, thus showing hopeful early results in preclinical studies [35].

Finally, it is imperative to treat every systemic manifestation of FD. A strict follow-up performed by an expert multidisciplinary team is essential, and preventive measures, such as administering antithrombotic agents for stroke prevention, are crucial to avoid severe events that can significantly compromise prognosis [29].

## 2. Materials and Methods

An advanced literature search was performed on the PubMed central database, using “Fabry disease” or “Anderson-Fabry disease” and a combination of other keywords referring to ophthalmology with Boolean operators AND and OR. Specifically, we searched for: “eye”, “cornea”, “cornea verticillata”, “conjunctiva”, “vascular”, “lens”, “lens opacity”, “cataract”, “retina”, “retinal vasculature”, “enzyme replacement therapy”, “in vivo confocal microscopy”, “OCT”, “OCT-A”, “OCT angiography”, and “mitochondrial micro RNAs” as keywords. Articles were sourced from PubMed and Embase, with additional searches performed using different keyword combinations at the authors’ discretion. Studies available in these databases from inception up to May 2024 were included. We reviewed a total of 93 papers and included only the most relevant for each section. We identified them by reviewing abstracts, and full texts of pertinent papers were read. In cases where multiple studies reported similar findings, the most recent research was generally selected for the review.

## 3. Ophthalmological Features

**Figure 2:**
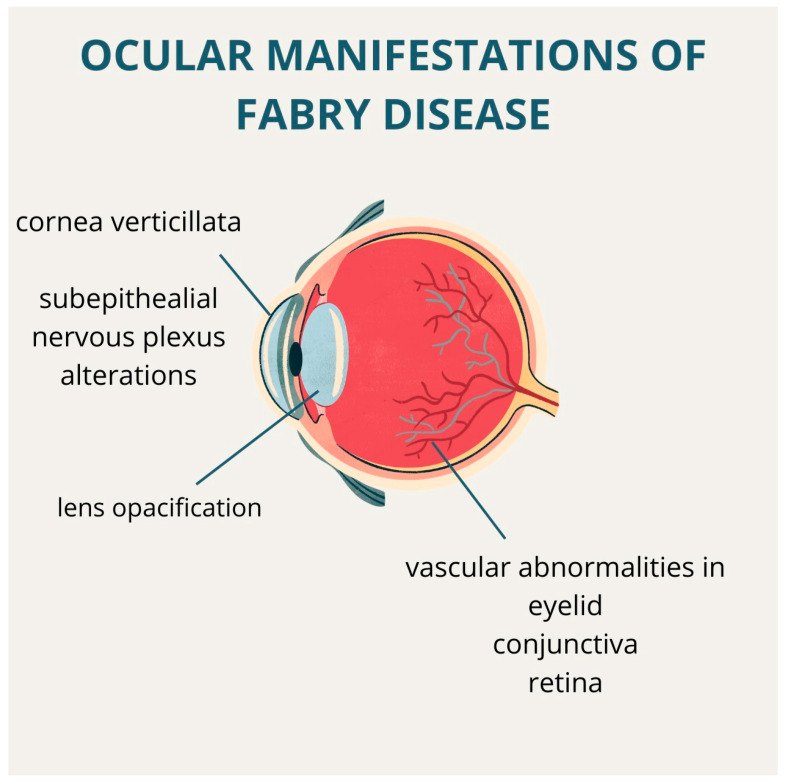
Schematic representation of the three main categories of ocular manifestations in Fabry disease: corneal alterations, vascular abnormalities, and lens opacification.

Fabry disease is often associated with ophthalmological signs, which can be categorized into three main groups: (1) corneal alterations, (2) vascular abnormalities, and (3) lens opacities. The p.M187R GLA mutation is linked to an increased prevalence of ocular signs, particularly retinal vessel alterations. These ocular manifestations correlate directly with the severity of the disease, as measured by the general, renal, and neurological sections of the MSSI [36] (Figure 2).

### 3.1. Cornea Verticillata

Corneal alterations can appear very early in life and have been detected in a 22-week fetus [37].

Cornea verticillata (CV) is the most common ocular finding, occurring in approximately 94% of classic-phenotype men and 88% of affected women [38].

It appears as a whorl-like corneal yellow-brown opacity in the inferior emicornea, resulting from lipid inclusion of unmetabolized Gb-3 [39].

**Figure 3:**
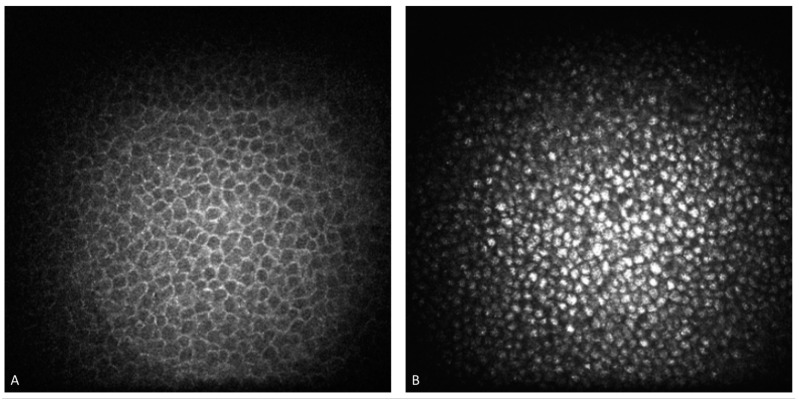
In vivo confocal microscopy examination of a healthy cornea (**A**) compared with a Fabry disease patient’s cornea (**B**), characterized by hyperreflective spots in the basal epithelium cells. The different appearance of corneal epithelial cells can help differentiate the etiology of cornea verticillata.

This distinctive sign has been recognized for over a century: following the first drawing of a corneal vortex pattern published in 1910 by Fleisher, in 1946, Gruber formally named the spiral pattern “cornea verticillata”. These inclusions fill the cells of the corneal basal epithelium layer and then spread to the basal membrane and extracellular spaces. The unmetabolized Gb-3 is stored in distended lysosomes, which can no longer function properly in intra- and extracellular transportation, leading to tissue dysfunction. In some cases, the lysosomal membrane fuses with the cellular membrane, releasing the Gb-3 load into the extracellular space. It is also possible that the lysosomal membrane fuses with other organelles, releasing Gb-3 into them. This storage results in hyperreflective microdots distributed in the basal epithelium, basal membrane, and sometimes in anterior stroma. The inclusions can appear in streaks, patches, or have a diffuse pattern. Gb-3 deposits can be detected by slit-lamp biomicroscopy examination or in vivo confocal microscopy, which has greater sensitivity in identifying the hyperreflective specks in the basal epithelium layer [39] Figure 3.

The macroscopic phenotype of cornea verticillata, visible during biomicroscopic examination, varies. The most common presentation is a whorl-like pattern, but other variations include patchy deposition opacities, vertical streams from the superior limbus, and diffuse epithelial haze [40].

Cornea verticillata is usually asymptomatic. Despite the opacities, the cornea maintains sufficient transparency to allow normal visual function. However, irregularities at the interfaces of the anterior and posterior corneal layers, which are the principal sources of corneal light scattering, can cause increased backscattering, leading to glare and reduced vision quality. Patients evaluated with contrast sensitivity function tests have indeed shown functional deficits [41].

Corneal densitometry values are also statistically significantly higher in all three depth levels analyzed in FD patients compared with healthy subjects, which could serve as a potential early diagnosis indicator [42].

Interestingly, a reduction in cornea verticillata has been noted after several years of ERT. A Danish study showed that corneal deposits decreased significantly in 13 out of 32 FD patients, becoming difficult to detect by slit-lamp examination after 10 years of follow-up [43]. These findings were corroborated by Shizuka Koh et al., who documented marked regression of corneal deposits after 16 years of ERT [44].

It is important to note that CV is not exclusive to FD. The primary cause of CV is the use of amphiphilic drugs, such as amiodarone. Differentiating between these two etiologies cannot be achieved by slit-lamp examination alone, but can be achieved by in vivo confocal microscopy (IVCM) [45,46]. This exam highlights differences between the two forms: in FD, the Gb-3 accumulation fills the entire cytoplasm with a regular pattern and homogeneous size, and the reflectivity is less pronounced compared to CV caused by amiodarone accumulation. Additionally, the subepithelial nerve plexus and endothelium are not affected by inclusions in FD [38,47].

The presence of CV can aid in the diagnosis, especially in non-classical and later-onset patterns, when associated with other symptoms. However, its absence does not exclude the disease. In some cases, the classic pattern of the disease does not carry corneal alterations, or alterations can be detected only through IVCM rather than by slit-lamp examination [39].

Finally, cornea verticillata may act as a prognostic biomarker for variations in retinal vessel density (VD) levels and the progression of the disease. The co-occurrence of cornea verticillata and elevated VD levels could be utilized as a diagnostic indicator for Fabry disease. However, due to inconsistencies in VD measurements across different studies, additional research is required to substantiate this hypothesis [48].

### 3.2. Subepithelial Nervous Plexus Alterations

IVCM examination of the corneal nerve plexus of FD patients shows a significant reduction in the length, number, and density of the nerve fibers, along with increased tortuosity, even in the absence of corneal deposits. Hyperreflective dots and alteration in the corneal nerve plexus may be detected on IVCM, even if no other signs and symptoms are evident. Therefore, it is important to consider this real-time and noninvasive tool for early diagnosis in patients at risk [39].

**Figure 4:**
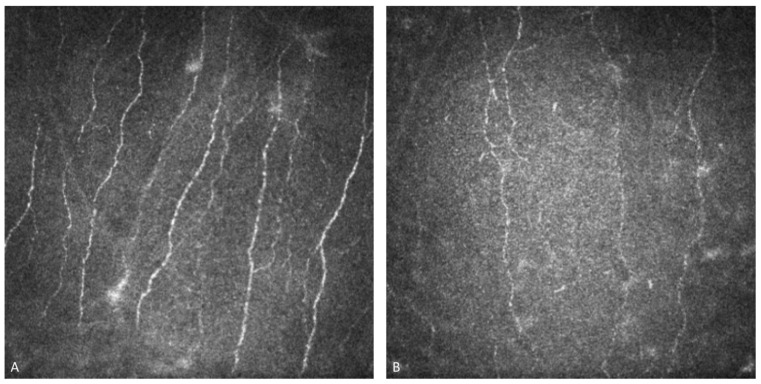
In vivo confocal microscopy examination of the sub-basal nervous plexus of a healthy cornea (**A**) compared with a Fabry disease patient’s cornea (**B**), characterized by a significant reduction in the length, number, and density of the nerve fibers, and by an increased tortuosity.

Corneal nerve fiber density in female and male patients who are treated with ERT is reduced compared with control subjects. Corneal nerve branch density is reduced in females not on ERT and in female or male patients on ERT compared to control subjects. Furthermore, corneal nerve length does not differ in FD females according to ERT status, but is significantly reduced in males on ERT compared with controls. These findings underscore the importance of using IVCM not only for early diagnosis but also for ongoing monitoring of corneal nerve health in FD patients, regardless of their treatment status [49] (Figure 4).

Variations in corneal nerve fibers in FD patients have functional implications. Corneal esthesiometry values, which measure corneal sensitivity, are significantly reduced in both male and female FD patients on ERT compared with control subjects. However, these values are not significantly reduced in female patients who are not on ERT, suggesting less severe nerve damage in women. This difference may reflect the milder progression of nerve damage in female patients with FD [50].

Moreover, the reduced corneal esthesiometry values in FD patients contribute to an increased incidence of dry-eye symptoms in FD patients, due to decreased tear secretion reflex stimulation. This highlights the importance of considering the impact of corneal nerve dysfunction on ocular surface health in FD patients [50].

It is also important to note that refractive surgery in FD patients tends to be unpredictable and often yields unsatisfactory outcomes. This unpredictability is due to instability of corneal anomalies. Moreover, the atrophy of nerve fibers predisposes the patient to dry eye. Currently, studies evaluating the safety and visual outcome of photorefractive keratectomy (PRK) and laser-assisted in situ keratomileusis (LASIK) in FD patients are unavailable, underscoring the need for caution in considering these procedures for individuals with FD [51].

### 3.3. Vascular Disorders

Vascular changes in FD are characterized by conjunctival and retinal vessel tortuosity, along with aneurysms of the conjunctival vessels, which are more frequently observed in the nasal bulbar conjunctiva. Notably, anomalies of conjunctival vessels are not associated with retinal vascular alterations [52].

These vascular changes have been detected in over 20% of male patients and result from the accumulation of glycosphingolipids in the cytoplasm of endothelial cells, thus creating loss of vascular wall resistance. This pathological process can compromise tissue perfusion and stimulate the release of vascular growth factors like vascular endothelial growth factors (VEGF) by affected cells [53,54].

Anecdotal reports of central retinal artery occlusion have been described, but no clear association between this condition and FD has been established [43,55].

#### 3.3.1. Eyelid Vascular Tortuosity

Another possible finding in FD patients is vessel tortuosity of the upper eyelid vessels, which tends to be more pronounced in male patients undergoing ERT. This feature should not be confused with blepharitis, as it lacks the typical signs and symptoms of eyelid inflammation, such as debris, redness, and foreign body sensation. Additionally, in FD, vessel tortuosity primarily affects the central part of the eyelid surface rather than the margin. Importantly, there is no evidence of functional impairment associated with vessel tortuosity in FD [56].

#### 3.3.2. Conjunctival Alterations

Conjunctival epithelial cells in FD can also exhibit alterations. These cells may present with irregular morphology and poorly distinguishable cell borders. IVCM may reveal hyperreflective material interspersed between areas of normal cellular architecture, which may be due to an increase in the autophagosome protein LC3, an indirect marker of the heightened presence of autophagic vacuoles [4].

The most common feature observed is the presence of roundish hyperreflective intracellular structures. Additionally, the tarsal conjunctiva may display papillary columnar reflective inclusions, indicative of further conjunctival involvement in FD [57].

#### 3.3.3. Retinal Vascular Alterations

Retinal vascular tortuosity and alterations are significant ophthalmological signs in FD, which can be effectively monitored using fundus oculi examination and fluorescein angiography. A recent study highlighted that patients with FD have narrower retinal vessels compared with healthy subjects. Specifically, the peripapillary vessel diameter was found to be 10.9% smaller in FD patients, with temporal vascular arcades 7.8% thinner and second-order collaterals 7.4% thinner than those in controls. This assessment was performed using retinography digital images of both eyes, analyzed with dedicated semiautomatic software. These findings suggest that a semiquantitative evaluation of retinal vessel diameter and tortuosity could serve as a valuable marker for tracking disease progression and assessing systemic vascular involvement in FD [58].

The advent of optical coherence tomography angiography (OCT-A), which leverages the reflective properties of blood cell motion, allows noninvasive evaluation of microvascular alterations [47,59,60]. Retinal vessel tortuosity in FD can be quantitatively assessed using three parameters, as outlined in a paper by Sodi et al. in 2013: the sum of angle metrics (SOAM), the product of angle distance (PAD), and the triangular index (TI). All three parameters have been found to be elevated in FD patients compared to controls [61].

In addition to tortuosity, patients with FD demonstrate a reduced deep capillary vascular density (dCVD) across all macular sectors, while superficial capillary vascular density (sCVD) and choriocapillaris blood flow remain relatively unaffected. Notably, the reduction in dCVD has been negatively correlated with the duration of ERT, suggesting that treatment is often initiated after significant vascular damage has already occurred due to late diagnosis. This emphasizes the importance of early intervention to prevent irreversible microvascular alterations [58].

Minnella et al., in a 2019 study, examined macular vascular features in FD patients using OCT-A to investigate macular impairment caused by vascular rarefaction and tortuosity. Their findings highlighted a rarefaction-like ischemic area within the retinal capillary plexuses, particularly in the deep capillary plexus. Additionally, the superficial capillary plexus exhibited increased vascular tortuosity. These vascular changes suggest the presence of subclinical ischemia, despite the preservation of visual function. To further investigate the macular function, patients underwent focal electroretinography (fERG). The results indicated a potential compromise in blood supply to photoreceptors and bipolar cells. However, these findings did not significantly correlate with the enlargement of the foveal avascular zone (FAZ) observed in these patients [62]. This discrepancy suggests that other factors, such as the impaired membrane trafficking due to GB-3 accumulation, common in lysosomal storage diseases, may contribute to compromise outer retinal function [63]. For these reasons, it is important to consider fERG a useful tool for assessing and identifying subclinical retinal alterations in FD. Additionally, OCT-A revealed an increase in vascular density, despite the observed capillary plexus rarefaction and enlarged FAZ [64]. This seemingly contradictory finding may be explained by the increased vascular tortuosity, which can influence OCT signal reflectance, potentially leading to an overestimation of vascular density.

Spectral domain OCT has enabled the detection of inner retinal hyperreflective foci (HRF), particularly in the parafoveal region. The quantitative score attributed to HRF has shown a significant correlation with the laboratory parameter of lyso-Gb3, which is commonly used to monitor FD. This correlation suggests that the HRF score should be further investigated as a biomarker for monitoring FD progression in clinical practice [65].

Until now, there has been no substantial evidence of neuroretina involvement in FD patients. However, a single case report by Wisely, C. E., et al. describes a patient with traction retinoschisis associated with a cystoid macular edema. Interestingly, this case describes the improvement in macular edema following ERT initiation, providing further evidence of ocular findings in FD improving with enzymatic treatment [66].

### 3.4. Lens Modifications

Lens opacities represent the third category of ophthalmological signs detected in FD. The most characteristic finding is the presence of linear translucent deposits of GL-3 within the lens epithelium, typically located along the suture lines near the posterior lens capsule, commonly referred to as “Fabry cataracts” [67,68].

Less frequently, FD patients may present with an anterior or subcapsular, wedge-shaped, cream-colored lens opacity. The clinical significance of these lens opacities, including whether their presence correlates with disease severity scores, is still unclear. Further studies are needed to determine their prognostic value in FD [69].

## 4. Discussion

The aim of this paper is to emphasize the critical role of ophthalmological signs in diagnosing and managing FD. Detecting ocular alterations is crucial, not only for making an early diagnosis but also for effectively managing the disease and its associated comorbidities, as well as understanding the patient’s prognosis. Including an ophthalmological evaluation in new guidelines would be highly beneficial. In pediatric patients, ophthalmological signs are particularly significant, as they can correlate with the severity of the disease. For instance, a study has shown that children with eye findings tend to have more severe disease, as measured by age-adjusted FOS-MSSI total scores, compared with those without eye findings (median, 0.5 versus −2.3; *p* < 0.001) [37].

In non-classical or later-onset clinical patterns, where typical symptoms may be absent, a definitive diagnosis can be challenging. In such cases, ophthalmological signs can guide the physician to a correct assessment. It is important to note that all ocular findings, given that the eye is an external organ, can be easily detected through non-invasive exams. These include slit-lamp examination, fundus ocular exam, IVCM, and OCT-A. These tools offer non-invasive and effective means of monitoring FD progression and aiding in its diagnosis, especially when other systemic symptoms may not be as evident.

An early diagnosis enables the timely initiation of ERT, the cornerstone of FD management. When started early, it significantly improves overall outcomes and reduces the likelihood of acute systemic events. Recent case reports and case series have proved that ocular alterations associated with FD can distinctly improve after several years of ERT. This indicates that early ERT not only prevents severe systemic events, reduces organ damage, and decreases mortality but also enhances visual outcomes for patients [43,44].

New approaches in FD diagnosis are represented by machine learning algorithms and ongoing studies investigating new biomarkers and their clinical relevance. AI technology holds promise for earlier diagnosis of Fabry disease by leveraging statistical patterns identified from extensive datasets of patients with the condition [70].

Another promising field of research involves exploring mitochondrial microRNAs (mitomiRs), which appear to be dysregulated in FD patients. These mitomiRs could potentially be used to monitor the response to therapy or even serve as therapeutic targets themselves [71]. The eye could be an accessible site for sample collection and analysis, and the role of microRNAs in ocular diseases is an up-and-coming line of research [72].

Given these insights, it is imperative that other healthcare specialists recognize the importance of referring patients to an ophthalmologist when FD is suspected. Ophthalmologists in turn should be aware of the critical role they play in the early initiation of treatment. Early intervention is not only essential for managing ocular symptoms but is also vital for preventing life-threatening complications and improving the overall prognosis of these fragile patients. This multidisciplinary approach ensures that FD is managed comprehensively, with the best possible outcomes for the patients.

## 5. Conclusions

In conclusion, ophthalmological exams play a crucial role in diagnosing and managing Fabry disease (FD). Ocular signs are valuable for early detection, especially in pediatric patients, where they can indicate disease severity. In non-classical or late-onset cases, these signs can guide diagnosis when systemic symptoms are absent. Non-invasive exams like slit-lamp and fundus exams, IVCM, and OCT-A allow for effective monitoring. Therefore, the ophthalmological assessment appears to be crucial in every step of the patient’s journey and should be included in up-to-date guidelines.

Starting enzyme replacement therapy (ERT) early improves outcomes, reducing systemic complications and enhancing ocular health. New research into biomarkers like mitochondrial microRNAs (mitomiRs) may further aid treatment. A multidisciplinary approach is essential, with prompt referrals and early intervention to optimize patient prognosis and quality of life.
